# Understanding the effect of loneliness on unemployment: propensity score matching

**DOI:** 10.1186/s12889-022-13107-x

**Published:** 2022-04-28

**Authors:** N Morrish, R Mujica-Mota, A Medina-Lara

**Affiliations:** 1 Health Economics Group, Institute of Health Research, University of Exeter Medical School, University of Exeter, Exeter, UK; 2 Academic Unit of Health Economics, School of Medicine, University of Leeds, Leeds, UK

**Keywords:** Loneliness, Unemployment, Working-age, United Kingdom, Bidirectional, Propensity score

## Abstract

**Background:**

Loneliness and unemployment are each detrimental to health and well-being. Recent evidence suggests a potential bidirectional relationship between loneliness and unemployment in working age individuals. As most existing research focuses on the outcomes of unemployment, this paper seeks to understand the impact of loneliness on unemployment, potential interaction with physical health, and assess bidirectionality in the working age population.

**Methods:**

This study utilised data from waves 9 (2017–19) and 10 (2018–2020) of the Understanding Society UK Household Longitudinal Study. Nearest-neighbour probit propensity score matching with at least one match was used to infer causality by mimicking randomisation. Analysis was conducted in three steps: propensity score estimation; matching; and stratification. Propensity scores were estimated controlling for age, gender, ethnicity, education, marital status, household composition, number of own children in household and region. Findings were confirmed in panel data random effect models, and heterogeneous treatment effects assessed by the matching-smoothing method.

**Results:**

Experience of loneliness in at least one wave increased the probability of being unemployed in wave 10 by 17.5 [95%CI: 14.8, 20.2] percentage points. Subgroup analysis revealed a greater effect from sustained than transitory loneliness. Further exploratory analysis identified a positive average treatment effect, of smaller magnitude, for unemployment on loneliness suggesting bidirectionality in the relationship. The impact of loneliness on unemployment was further exacerbated by interaction with physical health.

**Conclusions:**

This is the first study to directly consider the potentially bidirectional relationship between loneliness and unemployment through analysis of longitudinal data from a representative sample of the working age population. Findings reinforce the need for greater recognition of wider societal impacts of loneliness. Given the persisting and potentially scarring effects of both loneliness and unemployment on health and the economy, prevention of both experiences is key. Decreased loneliness could mitigate unemployment, and employment abate loneliness, which may in turn relate positively to other factors including health and quality of life. Thus, particular attention should be paid to loneliness with additional support from employers and government to improve health and well-being.

**Supplementary Information:**

The online version contains supplementary material available at 10.1186/s12889-022-13107-x.

## Introduction

Loneliness encapsulates the deficit between an individual’s desired and actual social relationships [1]. It is commonly described as ‘the feeling we get when our need for rewarding social contact and relationships is not met’ [2] and can be understood as the subjective or perceived experience of isolation or lack of social support. Loneliness can affect anyone, at any age, and in any circumstance [3]. A person may be lonely without being socially isolated [4] as loneliness incorporates not only the quantity but also quality of social interaction. Thus, while related, loneliness is distinguishable from social isolation and being alone. Loneliness in the adult population is associated with age, gender, sexual orientation, ethnicity, personality, and personal and familial circumstances involving marital status and socio-economic status, cohabitation, and health. Loneliness risk factors also include long-term illness, cognitive impairment, low self-esteem, reduced life-satisfaction, introversion, and life changing events such as bereavement, relocation, divorce, or undertaking of caring responsibilities [2, 5–10]. The current COVID-19 pandemic and resulting public health crisis has exacerbated the experience of loneliness [11] which had already been described as a ‘public health epidemic’ [12]. Furthermore, increased digitalisation in the way people work, shop, and seek healthcare as a result of both technological advances and COVID-19 means aspects such as individualism and low perceived social support will become ever more prevalent in society.

Health and social care research evidences a relationship between loneliness and health related outcomes, particularly amongst the elderly [13]. Prominent amongst health effects are cardiovascular disease, depression and mortality, while the overall health impact of loneliness is considered of comparable magnitude to that of smoking, and greater than both obesity and physical inactivity [14]. Prevention of loneliness may thus reduce health related costs. This avoidable health related cost of loneliness over a 10-year period in the elderly population was estimated to be in excess of £1,700 per person, or greater than £6,000 for older people experiencing loneliness ‘most of the time’ [15]. While existing research is concentrated in the older population, loneliness can be experienced at any age with its detrimental impact on health and well-being present across the life course [13]. Recent research suggests young and middle-aged adults self-report loneliness most frequently, with the highest prevalence in younger people and the lowest in the elderly [5]. In the working age population, the impact of loneliness is wide-ranging, encompassing health, economic, employment and education outcomes [16]. In 2017 the UK cost of loneliness to employers was estimated to be £2.5 billion per annum [17]. This estimate includes the effect of loneliness on sickness absence, days lost to carers leave, lower productivity and lower staff retention. This estimate is likely to fall short of the true cost owing to the overlapping effect of conditions such as social isolation and depression, and the additional effect of loneliness on unemployment. In addition to restricting economic growth, unemployment, like loneliness, has a detrimental impact on mental health, overuse of healthcare resources, suicide, substance abuse and poorer quality of life [18–25].

This paper focuses on loneliness in the working age population, evaluating the human cost of unfulfilled potential in the relationship between loneliness and unemployment. It also considers the impact of life satisfaction and work limiting physical health in the relationship, distinguishing between sustained and transitory duration of loneliness. A positive correlation between loneliness and unemployment has been evidenced by a number of studies summarised in a recent review [26]. However, in these studies the relationship between loneliness and unemployment often arises as an incidental finding. Furthermore, causal analysis is rare and often limited to investigating the impact of unemployment on loneliness, which is a more established and better-understood direction of effect [27–29]. However, reports from recent longitudinal studies suggest loneliness predicts subsequent work disability and higher mid-life unemployment across Europe [30, 31]. Additionally, evidence from a British study of young adults found lonelier individuals to be more likely unemployed, less prepared for the job market, and report lower work-related optimism at age 18 [16]. Thus, there is emerging evidence of a bidirectional effect regarding loneliness and employment outcomes [26]. The causal impact of loneliness on employment outcomes suggested by this research could motivate and guide home working policies and social prescribing to improve workforce well-being.

Recent studies have used propensity score matching to analyse causal relationships in health, loneliness, and occupation status [32–39]. This method has been preferred over instrumental variable frameworks which present frequent challenges in identifying an appropriate instrument. There is limited generalisable evidence on causal relationships as studies considering loneliness and unemployment have been largely based on cross-sectional data [8, 16, 28, 29, 40–42]. This study expands on the methodological paper by Buecker and colleagues who used propensity score matching to evaluate loneliness surrounding major life events [37], and adds to the literature on the impact of loneliness in society. It performs both cross-sectional and longitudinal analysis to consider the causal effect of current, past, sustained, and recent onset loneliness on employment status.

## Methods

### Data

This study utilised data from the Understanding Society UK Household Longitudinal Study [43]. This longitudinal survey collects data through a self-completion online survey or through face-to-face interviews with UK adults aged 16 and above. It aims to study the effect of social, economic and policy change on the well-being of the UK population. Data from wave 9 (2017–19) and wave 10 (2018–2020) were used in this study as loneliness was only included as a variable in data collection from wave 9 onwards. Analysis was restricted to the working age population, with those aged over 65 excluded from the sample.

Loneliness was assessed though the direct question ‘how often do you feel lonely?’. Responses were recorded using three levels: hardly ever/never, some of the time, often. Missing responses for loneliness were excluded from analysis and loneliness was transformed into a binary variable. Loneliness was considered present where an individual reported feeling lonely often. Unemployment was assessed by the question ‘which of these best describes your current employment situation?’ Employment status was described across 12 categories and transformed into a binary variable, being unemployed or employed, for analysis. The data did not differentiate between unemployed individuals looking for work and those not looking for work. Therefore, in this study unemployment includes both individuals experiencing involuntary and voluntary unemployment. Individuals in full-time education, or with ambiguity on work status were excluded from analysis (see Table A1, Appendix 1). Those permanently sick or disabled were considered unemployed but were omitted from the data in sensitivity analysis given the uncertain extent to which long-term sickness or disability were due to loneliness in our sample.

### Econometric analysis

The analysis was based on propensity score matching (PSM) [44–47]. PSM enables causal inference from observational data by mimicking experimental randomisation [48] of observed individuals to treatment and control groups, defined respectively by their having and not having a relevant condition, as individuals are matched across the two groups on the basis of the propensity score. The propensity score is defined as the latent probability of exposure to the condition and encompasses all selected confounders in a single value bringing simplicity and reducing bias in estimation of a treatment effect as confounders are balanced between treatment groups [47]. The use of PSM in this study builds on recent research in health, loneliness, and occupation status where PSM is used to balance covariates, minimise potential confounding and emulate randomisation [32–37]. The main purpose of this study was to use PSM to estimate the treatment effect of loneliness on unemployment. A secondary objective was to estimate the effect of unemployment on loneliness, conducted as exploratory analysis. The use of PSM rather than regression adjustment provides greater flexibility, particularly in analysis of the effect of unemployment on loneliness where exposure to unemployment (risk factor) is common but loneliness (outcome) reported less frequently [48]. PSM also mimics a randomised trial where participant characteristics do not dictate exposure and thus reduces selection bias. Finally, PSM demands overlap in propensities across exposure groups to ensure a sufficient number of similar individuals in the treated (or exposed) and untreated (or non-exposed) groups are included in the analysis. This ensures a representative comparison between exposed and unexposed as a whole, rather than focus on the upper and lower bounds or best and worst case scenarios [48].

Data were analysed in STATA/SE 16.0. PSM was conducted in three steps: estimation of propensity score; matching; and stratification. First, given the shortcomings of the linear model, particularly with skewed data, the propensity score was estimated using a probit regression [44]. Covariates were selected based on known risk factors for loneliness. Proposed variables were confirmed by comparison with studies considering the effect of loneliness on daily outcomes [49, 50] and variable availability in the survey data. The final selection of covariates included: age, gender, ethnicity, education, marital status, household composition, number of own children in household and region. Consistent with recent PSM studies in health and employment [38, 39], square terms were not used for matching. The propensity score (e(x_i_)) is the individual probability of exposure (z = 1, in this case loneliness) vs non-exposure (control (z = 0), in this case no experience of loneliness), given a number of observed characteristics (x_i_) [51]:

$$e({x}_{i}) = Pr(z=1|{x}_{i})$$

where x_i_ is a vector of covariates including age_i_, gender_i_, ethnicity_i_, education_i_, marital status_i_, household composition_i_, number of own children in household_i_ and region_i_ for individual i in the sample.

The propensity score provides an indicator of similarities between individuals by combining a set of covariates (x_i_) into a single dimension (scalar) thus facilitating their comparison. Covariates included in the model simultaneously affect the treatment decision and the outcome under consideration, and are unaffected by participation in the treatment decision or the anticipation of participation [44]. This allows us to mimic a randomised trial where the participant characteristics (covariates) do not dictate treatment allocation in the sample selected for analysis using PSM. However, a balance had to be struck between excluding only covariates unrelated to the outcome [44, 52], while also avoiding over-parameterisation which exacerbates the support problem and increases variance [44, 53]. Covariate selection was also informed by existing PSM analyses of loneliness [33, 34, 37], however these focussed on loneliness as an outcome and therefore not all cited covariates were considered appropriate at this stage.

Secondly, individuals were matched based on the aforementioned covariates across exposed and non-exposed groups, according to their propensity scores. Loneliness was the exposure and unemployment the outcome. Matching was based on the nearest-neighbour method as used in previous studies utilising PSM with loneliness as an outcome [33, 37]. Nearest-neighbour matching ensured a matching partner for an exposed individual was selected from the comparison group based on the closest propensity score. As fewer respondents had experience of loneliness (exposed) than no experience of loneliness (unexposed) each individual in the exposed group was matched with at least one individual from the unexposed. Meanwhile a single match was found for each of the unexposed individuals. Respondents were matched to their closest neighbour. There was no maximum distance for matches as no caliper was specified in order to retain all observations and ensure no data were lost. This approach was consistent with recent PSM study in loneliness and major life events [37]. Finally, stratification was conducted by comparing mean outcomes across people in different groups (strata), exposed vs non-exposed (or treated vs non-treated), with similar propensity scores.

Following the process of matching based on age, gender, ethnicity, education, marital status, household composition, number of own children in household and region, the Average Treatment Effect (ATE) comparing outcomes between those with any experience of loneliness to no experience of loneliness was estimated [51]:

$$ATE=E\left({r}_{1}\right)-E({r}_{0})$$

where E(r_j_) denotes the expected probability of outcome r (unemployment) in group j. Here j = 1 for individuals with any experience of loneliness in waves 9 or 10; j = 0 otherwise (no experience of loneliness).

The exposure effect of loneliness on unemployment was tested from both a cross-sectional and longitudinal perspective using waves 9 and 10 of the Understanding Society dataset. This allows comparison to existing cross-sectional studies while additionally expanding findings to achieve causal inference. In the cross-sectional analysis, each wave was considered independently estimating whether present day loneliness contributes to current unemployment in wave 9 (model 1) and wave 10 (model 2). In longitudinal analysis, data from both waves 9 and 10 of the dataset were utilised to understand the ATE of loneliness at any time point (j = 1), when compared to no experience of loneliness (j = 0), on unemployment in wave 10 (model 3). In all models exposure to loneliness was compared to a control of no experience of loneliness. Statistical significance was tested by *p*-values and 95% confidence intervals, and the null hypothesis rejected where at least 5-percent statistical significance was not achieved.

### Model heterogeneity and balance

Heterogeneous treatment effects were assessed in cross-sectional data using the matching-smoothing method. Matching-smoothing retains individual-level information before making cross-individual comparisons and so overcomes the assumption of homogeneity within strata in detecting heterogeneous treatment effects [54]. Results are represented by a plot of exposed and non-exposed individuals against a continuous propensity score before a local polynomial fit of degree 1 (local-linear smoothing) is fitted to the matched difference yielding a pattern of treatment effect heterogeneity [54]. Additionally, the two cross-sectional models were combined in single panel data probit random effect models allowing for contemporaneous effects while adjusting for unobserved heterogeneity across individuals. Random effect models were run on the propensity score matched sample using panel data probit regression. As no caliper was specified all respondents had a match and so random effects were run on the full sample. The model was first run with only loneliness included as a covariate then run again including the additional aforementioned covariates:

$${Y}_{it}+1={X}_{it}\beta +\alpha +{\varepsilon }_{it}$$

where Y_it_ is the unemployment outcome; X_it_ is a vector of covariates including loneliness_it_, age_it_, gender_it_, ethnicity_it_, education_it_, marital status_it_, household composition_it_, number of own children in household_it_ and region_it_ for individual i in the sample; and α is the unobserved normally distributed mean zero random effects varying across individuals but not waves, and ε_it_ is a mean zero normally distributed random error varying across individuals and waves. Age was included as a continuous variable in years; female, white ethnicity, lower than higher education, married/civil partnership, presence of ≥ 2 adults in household, presence of ≥ 1 children in household, Southern England residence, loneliness and unemployment were coded as binary. Further detail of this coding is provided in Table A2, Appendix 1.

Random effects were then run using baseline propensity score as an importance weight in panel data probit regression. Fixed effect methods were also explored however not included in this paper since only a few individuals changed employment status. Covariate balance was assessed through analysis of standardised differences and variance ratios across raw and matched data. Balance was signified by standardised differences less than 0.1 and variance ratios close to 1 [48].

### Sensitivity analysis

Sensitivity analysis was first conducted by reclassifying loneliness to include those individuals who reported experiencing loneliness ‘sometimes’. Unemployment was reclassified in sensitivity analysis by excluding those who were permanently sick or disabled at baseline from the analysis. Analysis was also run excluding early retirees from the sample. The addition of covariates for life-satisfaction and physical health limitations was also explored. Overall life satisfaction was measured by a single item question with seven response levels ranging from completely dissatisfied to completely satisfied. Physical health was measured with respect to how much current physical health limited the amount of work conducted by an individual in the last 4 weeks. While neither were included as a covariate in previous studies, they are risk factors for loneliness [7, 9, 10, 55] and their impact was assessed in sensitivity analysis. Furthermore, the inclusion of work limiting physical health facilitated some understanding of the potential for health-related interaction effects. While included in previous studies, it is conceivable that both loneliness in the baseline period and incident unemployment in the intervening period may influence marital status and household composition. These variables are more likely to affect than be affected by loneliness, however for robustness they were excluded in sensitivity analysis.

### Subgroup analysis

Subgroup analysis was conducted on the matched sample to understand the impact of change in loneliness over time (between waves 9 and 10). Indicator variables were created to distinguish between sustained loneliness across the two waves, the onset of loneliness in the intervening period, the change from experiencing to not experiencing loneliness, and no experience of loneliness in either time point. These variables were included in a probit regression model with wave 10 unemployment as the outcome (model 4a) and latent linear index:

$$Y=a+bD1+cD2+eD3+e$$

where D1 = 1 if sustained loneliness, 0 otherwise; D2 = 1 if onset of feeling lonely, 0 otherwise; D3 = 1 if moving from experiencing loneliness to not experiencing loneliness, 0 otherwise; and e is the error term. The constant a represents the expected outcome for the reference group without any feeling of loneliness throughout the period of analysis (i.e. D1 = D2 = D3 = 0). A dummy variable for ‘never’ feeling lonely was not included thus avoiding the dummy variable trap. This analysis was also modelled including all aforementioned baseline covariates (model 4b) to further explore potential heterogeneity. Covariates were coded as in the random effects models (Table A2, Appendix 1) to aid interpretation of coefficients.

Additionally, analysis of change in employment status was conducted in the subgroup of individuals employed in wave 9 to eliminate the contemporaneous impact of unmeasured background confounders. Probit regression of whether the respondent became unemployed in wave 10 or not as a function of loneliness in wave 9 was conducted. Analysis was first undertaken with only wave 9 loneliness included as an independent variable (simple model) and then repeated including covariates at baseline (full model). Again, variables were coded as in Table A2, Appendix 1. Finally, subgroup analysis considered the difference in outcomes across male and female respondents, and across age groups.

### Exploration of bidirectionality

Additional exploratory analysis was conducted with unemployment treated as the exposure to consider the reverse impact of being unemployed on experience of loneliness. PSM analysis evaluated the impact of unemployment in wave 9 and/or wave 10 on the outcome of loneliness at wave 10. Furthermore, subgroup analysis on the matched sample revealed the differential impact of sustained unemployment, becoming unemployed, and becoming employed on loneliness, relative to being employed in both waves. A covariate for general health was included in the main model given the potential association of health outcomes and unemployment, while covariates for household composition and number of own children in the household were omitted, in line with existing PSM research on the effects of unemployment [34, 37]. Thus, individuals were matched based on age, gender, ethnicity, education, marital status, region of residence, and health at baseline.

## Results

### Descriptive analysis

Descriptive statistics for the sample analysed are presented in Table 1. Categories are grouped to ease interpretation with full breakdown available on request. Data provided 19,566 observations in wave 9 and 18,833 in wave 10. Surveyed individuals in each wave had a mean age of 44 (SD = 13, range 16–65) with 54% female respondents. The majority (82%) of the sample was of white background followed by 11% of individuals with Asian ethnicity. Just under half of the sample (wave 9 = 45%; wave 10 = 46%) had a higher degree or equivalent, while 16% (waves 9 and 10) reported attaining no educational certificate. Most were married or in a civil partnership (54%) while around 12% were separated, divorced, or widowed. Two-thirds of the sample had none of their own children living in their household while three-quarters of households included at least one couple. In each wave 42% of respondents lived with children while only 32% reported having their own children in their household suggesting around 10% of respondents lived with children who were not their own. Over 40% of respondents resided in the South of England (East of England/London/East/West) with sample representation from all regions of the UK. In wave 9 loneliness was experienced ‘never or hardly ever’ by 62%, ‘some of the time’ by 29% and ‘often’ by 8% of individuals, while in wave 10 these figures were 60%, 32% and 8% respectively. Most respondents were employed in waves 9 and 10 (83% and 84%, respectively).

**Table 1:** Descriptive statistics

	**Wave 9 (*n* = 19,566)**	**Wave 10 (*n* = 18,833)**
**Age 16–65, mean(SD)**	43.51 (13.24)	43.69 (13.26)
**Gender, n(%)**		
Male	9,047 (46.24)	8,669 (46.03)
Female	10,519 (53.76)	10,164 (53.97)
**Ethnicity, n(%)**		
White background	16,035 (81.95)	15,446 (82.02)
Mixed background	431 (2.20)	438 (2.33)
Asian background	2,147 (10.97)	2,052 (10.90)
Black background	849 (4.34)	792 (4.21)
Other ethnic group	104 (0.53)	105 (0.56)
**Education, n(%)**		
Higher degree or equivalent	8,803 (44.99)	8,693 (46.16)
A/AS level or equivalent	2,231 (11.40)	2,131 (11.32)
GCSE/O level	4,176 (21.34)	3,898 (20.70)
Other school certificate	1,184 (6.06)	1,097 (5.82)
None of the above	3,171 (16.21)	3,014 (16.00)
**Marital status, n(%)**		
Single, never married/civil partnership	6,578 (33.62)	6,292 (33.41)
Married/civil partner	10,587 (54.11)	10,244 (54.39)
Separated/divorced/widowed	2,401 (12.27)	2,297 (12.20)
**Household composition, n(%)**		
1 adult no children	2,343 (11.97)	2,246 (11.93)
1 adult with child/children	653 (3.34)	604 (3.21)
Couple no children	4,482 (22.91)	4,239 (22.51)
Couple with child/children	5,092 (26.02)	4,891 (25.97)
2 or more adults, no couples, no children	1,352 (6.91)	1,305 (6.93)
2 or more adults, no couples, 1 or more children	720 (3.68)	726 (3.85)
3 or more adults, at least 1 couple, no children	3,124 (15.97)	3,078 (16.34)
3 or more adults, at least 1 couple, 1 or more children	1,800 (9.20)	1,744 (9.26)
**Number of own children in household, n(%)**		
0	13,208 (67.50)	12,784 (67.88)
1	2,814 (14.38)	2,660 (14.12)
2	2,743 (14.02)	2,630 (13.96)
3 or more	801 (4.09)	759 (4.03)
**Region, n(%)**		
North (East/West/Yorkshire and the Humber)	4,525 (23.13)	4,439 (23.57)
Midlands (East/West)	3,184 (16.27)	3,057 (16.23)
South (East of England/London/East/West)	8,301 (42.43)	7,907 (41.98)
Wales	1,102 (5.63)	1,043 (5.54)
Scotland	1,476 (7.54)	1,463 (7.77)
Northern Ireland	978 (5.00)	924 (4.91)
**How often feels lonely, n(%)**		
Hardly ever or never	12,196 (62.33)	11,342 (60.22)
Some of the time	5,744 (29.36)	5,965 (31.67)
Often	1,626 (8.31)	1,526 (8.10)
**Current economic activity, n(%)**		
Employed	16,319 (83.40)	15,734 (83.54)
Unemployed	3,247 (16.60)	3,099 (16.46)

Education had the highest proportion of missing data at around 11% while most variables had less than 1 percent missing. There were no missing data in gender, household composition or number of children in the household. Participants with missing data or aged outside the 16–65 range were dropped from analysis (8,301 in wave 9; 7,418 in wave 10). In wave 9 data on loneliness were incomplete for 1,943 respondents and employment status incomplete for 33. For wave 10 these were 1,533 and 39 individuals, respectively.

### Impact of loneliness on unemployment

Results depicting the impact of loneliness on unemployment can be seen in Table 2. Cross-sectional PSM analysis, in both waves 9 (model 1) and 10 (model 2), found current experience of loneliness when compared to no experience of loneliness in the same wave had a positive average treatment effect (ATE) on the probability of being unemployed. This ATE was larger in wave 10 at 0.196[95%CI: 0.166,0.227] than wave 9 at 0.160[0.129,0.191]. Thus, findings suggest feeling lonely raises the likelihood of current unemployment by at least 16 percentage points, or up to 19.6 percentage points. Causal inference was expanded through longitudinal analysis (model 3). Experience of loneliness at any or both time points has an ATE of 0.175[0.148,0.202] on follow-up (wave 10) unemployment. This suggests an experience of loneliness in the two-wave period, leads to a 17.5 percentage point effect on the probability of unemployment, as compared to an individual with no experience of loneliness in either wave.

**Table 2:** Propensity score matching results

Model	Observations	ATE	SE	P >|z|	95%CI
Model 1	19,566	0.160	0.016	0.000	[0.129,0.191]
Model 2	18,833	0.196	0.016	0.000	[0.166,0.227]
Model 3	15,675	0.175	0.014	0.000	[0.148,0.202]

Matching-smoothing showed evidence of some heterogeneous treatment effects across propensity scores, particularly in wave 9, suggesting some respondents may be more vulnerable to loneliness and associated outcomes (Figure A1, Appendix 1). Additionally, both random effect models showed a contemporaneous effect of loneliness on unemployment is robust to adjusting for unobserved heterogeneity across individuals. No groups were dropped from matched sample analysis as a nearest-neighbour match was found for all observations. Random effect models indicated loneliness to have the largest impact on unemployment in the full model including all covariates with coefficient β = 1.158[0.997,1.319] when run on the matched sample, and β = 1.070[0.557,1.583] with propensity score weights (Table A3, Appendix 1). Besides loneliness, we observed ethnicity (non-white), education (those without a higher degree), and number of own children in the household (at least one child) to have the greatest effect on unemployment, as identified by the full model presented in Table A3.

Detail on raw and matched balance for models and covariates are provided in Appendix 2. Table A4 demonstrates how nearest neighbour matching maximised the number observations available when compared to the raw format. Table A5 shows that overall balance was obtained with the chosen covariates since for each model standardised differences were below 0.1 in the matched sample ranging from -0.033 to 0.050, compared to range -0.289 to 0.141 in the original sample. Variance ratios were overall closer to 1 in the matched sample (range 0.604 to 1.420) than the original (range 0.803 to 1.702). Finally Figure A2 illustrates how balance between treated (exposed) and untreated (unexposed) is improved in the matched sample.

### Sensitivity analysis

Results from sensitivity analyses are provided in Table 3 and discussed below.

**Table 3:** PSM sensitivity analysis

Sensitivity analysis	Model 1		Model 2		Model 3	
	**Observations**	**ATE [95%CI]**	**Observations**	**ATE [95%CI]**	**Observations**	**ATE [95%CI]**
Lonely sometimes or often	19,566	0.084*** [0.071,0.097]	18,833	0.091*** [0.077,0.104]	15,675	0.082*** [0.069,0.095]
Male	9,047	0.207*** [0.156,0.257]	8,669	0.170*** [0.123,0.217]	7,260	0.198*** [0.152,0.243]
Female	10,519	0.158*** [0.118,0.197]	10,164	0.173*** [0.137,0.209]	8,413	0.159*** [0.125,0.193]
Excluding permanently sick/disabled	18,751	0.083*** [0.052,0.114]	18,014	0.088*** [0.060,0.116]	14,898	0.062*** [0.037,0.087]
Including life satisfaction	19,544	0.101*** [0.052,0.150]	18,809	0.084*** [0.042,0.126]	15,646	0.126*** [0.088,0.164]
Including work limiting physical health	19,499	0.064*** [0.034,0.094]	18,769	0.071*** [0.041,0.100]	15,595	0.077*** [0.051,0.102]
Excluding marital status and household composition	19,659	0.181*** [0.153,0.209]	18,957	0.191*** [0.161,0.222]	15,819	0.166*** [0.139,0.194]

^*^p < 0.05, **p < 0.01, ***p < 0.001

### Reclassification of loneliness and unemployment

A decrease in ATE of around 50% was observed in all PSM models when including the response ‘some of the time’ in defining an individual with experience of loneliness. Sensitivity analysis excluding early retirees had only a minimal impact on the magnitude of effect with the impact of loneliness in fact increasing by around 13% (results available on request). Excluding those permanently sick or disabled from analysis, rather than categorising them as unemployed, saw a decrease in ATE of around 60% in all PSM models. For model 3, reclassification of loneliness revealed ATE 0.082[0.069,0.095], and re-categorising unemployment changed ATE to 0.062[0.037,0.087], both retaining statistical significance.

### Alternative covariates

Inclusion of life satisfaction as a covariate reduced the treatment effect magnitude by up to 57% with revised ATE 0.084[0.042,0.126] (model 2). Introduction of work limiting physical health at baseline as a covariate reduced the magnitude of the treatment effect by between 64% (model 1) and 77% (model 3), with model 3 presenting ATE 0.077[0.051,0.102]. Exclusion of marital status and household composition from the model for potential interdependence had only a small impact on ATE ranging from 5% reduction (model 3) to 13% increase (model 1) in ATE. In all cases, covariate changes neither impacted statistical significance nor direction of effect.

### Subgroup analysis

Subgroup analysis was conducted using probit regression on the matched sample. Model outputs are presented in Table 4. Sustained loneliness across waves 9 and 10 yielded the largest effect on unemployment with probit regression coefficient β = 0.734[0.627,0.841] when only loneliness was included (model 4a), and β = 0.804[0.688,0.919] in a model with all loneliness and demographic covariates (model 4b). A smaller effect was observed for individuals experiencing onset of loneliness or no longer feeling lonely with respective coefficients β = 0.460[0.350,0.570] and β = 0.481[0.375,0.588] in the simple model, or β = 0.642[0.523,0.761] and β = 0.568[0.454,0.683] in the full model (model 4b). Probit analysis of the occurrence of unemployment in wave 10 among the subgroup of individuals employed in wave 9 confirmed that loneliness increased the likelihood of unemployment in the next wave. Results are presented in Table 5.

**Table 4:** Subgroup probit regression results

Regressors	Model 4a Coefficient [95%CI] *n* = 15,675	Model 4b Coefficient [95%CI] *n* = 15,675
Constant	-1.099***[-1.125,-1.073]	-2.591***[-2.739,-2.444]
Sustained loneliness	0.734***[0.627,0.841]	0.804***[0.688,0.919]
Onset loneliness	0.460***[0.350,0.570]	0.642***[0.523,0.761]
Stop experiencing loneliness	0.481***[0.375,0.588]	0.568***[0.454,0.683]
Gender	-	0.012[-0.041,0.064]
Age	-	0.035***[0.032,0.037]
Ethnicity	-	0.168***[0.094,0.242]
Education	-	0.247***[0.193,0.301]
Marital status	-	-0.152***[-0.221,-0.084]
Household composition	-	-0.074[-0.153,0.005]
Number own children in household	-	-0.556***[-0.627,-0.485]
Region	-	-0.152***[-0.206,-0.097]

^*^p < 0.05, **p < 0.01, ***p < 0.001. All variables are binary (see Appendix TableA2)

Note: individuals in matched sample experiencing sustained loneliness *n* = 590, onset loneliness *n* = 612, stop experiencing loneliness *n* = 652

**Table 5:** Probit regression of unemployment in wave 10 in the subgroup of those employed at wave 9 (*n* = 13,386)

Regressors	Simple model Coefficient [95%CI]	Full model Coefficient [95%CI]
Constant	-1.834***[-1.876,-1.791]	-2.455***[-2.682,-2.229]
Loneliness	0.256***[0.114,0.399]	0.272***[0.124,0.420]
Gender	-	0.010[-0.074,0.095]
Age	-	0.017***[0.013,0.021]
Ethnicity	-	0.098[-0.019,0.214]
Education	-	0.025[-0.060,0.110]
Marital status	-	-0.107[-0.215,0.001]
Household composition	-	-0.003[-0.131,0.126]
Number own children in household	-	-0.387***[-0.497,0.278]
Region	-	-0.055[-0.141,0.031]

^*^p < 0.05, **p < 0.01, ***p < 0.001. All variables are binary (see Appendix TableA2)

Analysis was conducted separately for male and female subgroups to explore any potential heterogeneity by gender. In wave 9 a greater effect was observed in men than women, while in wave 10 the effect was slightly larger in females. Longitudinal analysis (model 3) indicated a greater ATE in men (0.198[0.152,0.243]) than women (0.159[0.125,0.193]). Additional exploratory analysis found direction and statistical significance to persist throughout working age with stronger effect in middle aged adults. The greatest impact of loneliness on unemployment was observed in individuals aged 46–55 with ATE 0.236[0.184,0.287] and the smallest at age 16–25 with ATE 0.116[0.068,0.165]. A complete breakdown by age group can be seen in Table 6.

**Table 6:** Propensity score matching across age groups

**Age**	**Obs**	**Impact of loneliness (w9 or w10) on unemployment in w10**
		**ATE [95%CI]**
16–25	1,596	0.116*** [0.068, 0.165]
26–35	2,706	0.131*** [0.084, 0.177]
36–45	3,525	0.171*** [0.115, 0.227]
46–55	4,230	0.236*** [0.184, 0.287]
56–65	3,618	0.160*** [0.090, 0.230]

^*^p < 0.05, **p < 0.01, ***p < 0.001

### Exploration of bidirectionality

Bidirectional exploratory analysis results are presented in Table 7. PSM analysis of longitudinal data comparing unemployment at baseline and/or follow-up to no experience of unemployment in either wave, had an ATE of 0.078[0.053,0.102]. The simple probit regression model revealed that sustained unemployment across waves 9 and 10 had a greater impact on loneliness (β = 0.553[0.478,0.628]) than either becoming unemployed (β = 0.373[0.222,0.525]), or becoming employed (β = 0.387[0.210,0.564]).

**Table 7:** Exploratory analysis: impact of unemployment on loneliness

**PSM (*n* = 15,661)**	**ATE [95%CI]**
Any unemployment (wave 9 and/or 10)	0.078***[0.053,0.102]
**Probit regression (*n* = 15,661)**	**Coefficient [95%CI]**
Constant	-1.545***[-1.579,-1.511]
Sustained unemployment	0.553***[0.478,0.628]
Become unemployed	0.373***[0.222,0.525]
Become employed	0.387***[0.210,0.564]

^*^p < 0.05, **p < 0.01, ***p < 0.001

Note: individuals in matched sample experiencing sustained unemployment *n* = 1,954, onset unemployment *n* = 464, stop experiencing unemployment *n* = 332

## Discussion

### Loneliness and unemployment

This study addresses the novel suggestion of a direct relationship between loneliness and unemployment. It finds both a positive treatment effect of loneliness on unemployment and unemployment on loneliness suggesting a bidirectional effect in the relationship between loneliness and unemployment, as hypothesised by existing research [26]. It particularly supports the lesser-studied impact of loneliness on unemployment and extends analysis across the working age population adding to the literature concerning the wide-ranging impact of loneliness [16]. Existing literature suggested this connection may arise from aspects such as reduced job search motivation and lower workplace performance in lonely individuals [16]. Longitudinal models indicate a larger impact of loneliness on unemployment than the reverse. While contrary to some existing research [26], this finding supports more recent studies evaluating the impact of loneliness on future work disability [30] and midlife unemployment [31]. This study also extends recent work by Buecker and colleagues who utilised PSM in the study of loneliness surrounding major life events [37], including the effect of employment status and job loss. The authors found job loss to cause change in loneliness, however movement from unemployment to paid work or re-employment did not trigger a change. Unfortunately, however, Buecker did not evaluate the potential for a reverse impact of loneliness on life events.

Through cross-sectional analysis, this study finds present day loneliness to increase current unemployment by 16.0 percentage points in 2017–19 (wave 9) and 19.6 percentage points in 2018–20 (wave 10), showing an increase in effect over time. This is consistent with research in loneliness describing it as a public health epidemic [12] with increasing prevalence over time, particularly in younger adults [56], and throughout COVID-19 [11]. While this finding may in part have been influenced by the COVID-19 pandemic, which saw an increase in experience of loneliness, this is likely marginal given only a short period of the pandemic is covered by the data, which spanned January 2018 to June 2020.

Longitudinal analysis suggests that experiencing loneliness for at least one time point, when compared to those with no experience of loneliness during the two-wave period, increases the chance of unemployment at follow-up by 17.5 percentage points. Thus prevention, or reduction, of loneliness has the potential to decrease this excess unemployment. Meanwhile, random effect models provided further evidence that previous experience of loneliness influences future unemployment again suggesting benefit to tackling loneliness. However, while loneliness remains a significant predictor of unemployment in the random effect models, there is some evidence of heterogeneous treatment effects, reinforced across propensity scores in working adults, highlighting the need for further research into patient sub-groups at risk of loneliness and related outcomes.

Subgroup analysis through probit regression revealed the greatest impact arises from a sustained experience of loneliness. There was however also an effect of exposure to transitory loneliness as loneliness onset was seen to impact unemployment outcomes. This suggests there also exists an immediate effect of loneliness onset, confirming findings from cross-sectional analysis of a relationship between present day loneliness and unemployment. Conversely, an effect is also observed for those experiencing loneliness in wave 9 but no longer feeling lonely in wave 10 suggesting there may also be a residual effect of previous loneliness on current unemployment and thus prevention rather than treatment may be the most productive approach to preventing detrimental effects of loneliness. These more isolated effects of transitory changes in loneliness and unemployment require further research, particularly in the light of the COVID-19 pandemic and rising onset of both loneliness and unemployment.

Study findings were robust to revising the definition of loneliness to encompass the statement of feeling lonely ‘some of the time’. Magnitude of the impact of loneliness on unemployment decreased with inclusion of the less frequently lonely. This implies that more severe experience of loneliness has a far greater effect on employment outcomes, as suggested in existing literature [41, 55, 57], and so is of greater concern to society, policy, and practice. An effect is observed in both male and female sub-populations with greater magnitude largely present in men, consistent with the societal view of them being the primary earner. Meanwhile, loneliness is seen to influence unemployment throughout working life with prominence in middle age, a finding that warrants future research.

Little change was observed following exclusion of marital status and household composition as covariates. This suggests that while there is potential for these variables to be associated with the treatment or outcome, their inclusion in the model does not change the interpretation of findings. Inclusion of a covariate for life satisfaction reduced the magnitude of treatment effect, with the greatest change observed in the cross-sectional analysis of wave 10 data suggesting recent change in the interaction between loneliness and life satisfaction, which could in part have been due to the impending COVID-19 outbreak. However, as mentioned above, the pandemic is only partially covered by the data and so there is a need for further research. Excluding individuals who were permanently sick or disabled, and including a covariate for work limiting physical health each reduced the treatment effect across all three models. This illustrates an interaction effect with well-being and health status, as physical health status can account for some of the magnitude in effect. Excluding the long-term sick may on the one hand reduce the risk of bias in attributing impact from unrelated long-term illnesses on unemployment, while on the other hand increases the risk of underestimating the impact of loneliness on unemployment that occurs through long-term sickness. Overall, these findings suggest the impact of loneliness on unemployment is exacerbated by interaction with physical health outcomes. However, the robustness of the relationship to adjusting for physical health also suggests the effect of loneliness on unemployment is independent of these health-related factors.

Considering bidirectionality, unemployment at any time point is observed to increase feelings of loneliness at follow-up by 7.8 percentage points when compared to no experience of unemployment. This is smaller in magnitude than the less commonly studied effect of loneliness on unemployment. Inclusion of an income covariate reduced the effect of unemployment on loneliness consistent with the idea that income is on the causal path from unemployment to loneliness. Probit regression revealed sustained unemployment across both waves presents the greatest effect on loneliness adding to the literature on detrimental effects of long-term unemployment [19].

### Strengths and limitations

This study provides insight into the lesser-studied impact of loneliness on unemployment through cross-sectional and longitudinal analyses. Thus, evidence is established for both directions suggesting bidirectionality in the relationship between loneliness and unemployment. Although the Understanding Society dataset provides a large and inclusive sample, the data did not allow distinction between ‘unemployed looking for work’ and ‘unemployed not looking for work’.

PSM provides innovative insight into the relationship between loneliness and unemployment. Findings reported herein are based on random effect models, which have the limitation of assuming that covariates are independently distributed whereas they may in fact be related to the residual. Causal analysis could be strengthened and the potential for simultaneous equation bias avoided through additional methods such as instrumental variable (IV) analysis [58]. However, choosing an appropriate instrument is not always possible and, for this study, we were unable to identify a suitable instrument. We did conduct exploratory IV analysis to address potential reverse causality with marital status selected as an instrument for loneliness given the correlation identified in existing research [59, 60]. While acknowledging that it may fail the requirement of being independently associated with unemployment, IV estimates were of the same direction with average marginal effects and standard errors up to three times greater than the PSM ATE estimates, thus reinforcing our finding that greater loneliness increases unemployment (details are available from the authors). We considered but ultimately decided against conducting difference-in-difference analysis to adjust for potential background confounding given our short panel with two waves of current loneliness data available and no information on prior experience of loneliness prevented testing for the key common trends assumption for identification. Future studies may be able to investigate this question using longer data panels.

Although PSM methodology has limitations, such as potential for imbalance and unobserved confounding, these have been addressed as follows. PSM can suffer from imbalance when compared to a randomised experiment [61]. This was mitigated by the large sample size available from the Understanding Society dataset, and balance checks conducted for each model specification to ensure improvement from matching. In this study, nearest-neighbour matching ensured a match was found for each individual based on the closest propensity score and therefore no observations were lost. As in recent PSM studies in loneliness [37], caliper was not specified and so proximity of matches between individuals is unclear. However, given the unequal distribution of exposed and unexposed persons, this was preferred to a narrow caliper specification in order to maximise the sample size and number of matches found.

In any propensity score analysis, there is potential for bias from unmeasured confounders. This is mitigated by including a large number of covariates in the propensity score. Although, sexual orientation and self-esteem have been identified as risk factors for loneliness [2, 6, 7, 10] they were neither available in the dataset nor included in existing PSM studies of loneliness. Personality indicators were also not available in the dataset. While work limiting physical health indicators were included in sensitivity analysis, other health related information were not analysed given their subjectivity and to avoid including noise from non-confounder variables in the analysis. Findings did however suggest an interaction with physical health and so further research in this area would be beneficial. Finally, socioeconomic status was not directly included in this study. Instead, elements of socioeconomic status such as education, region, and household composition were included in this analysis. Inclusion of further socioeconomic variables may rather have led to over parameterisation. Finally, as noted in previous work utilising propensity scores to evaluate loneliness [33], we cannot dismiss that some associations may be due to construct or measurement overlap, particularly with related concepts such as social network. However, in response, it is important to note that loneliness is a distinct phenomenon when compared to related concepts such as social isolation [4]. Overall, we consider our covariate selection reflects both risk factors for loneliness and existing PSM studies.

### Policy implications and future research

This research suggests a need for greater recognition of the wider societal impacts of loneliness in the working age population, extending those identified in health. Given the persisting and potentially scarring effects of both loneliness and unemployment evidenced in this study and across existing literature [62–65] prevention efforts should be directed at both experiences. Existing research, while limited in causal inference, suggests particular focus should be placed on younger middle-aged adults where the strength of the relationship between unemployment and loneliness is greatest [26, 66]. In younger populations there is also existing evidence that loneliness not only impacts rate of unemployment but is related to lower optimism and job market preparedness [16]. This study however shows the impact of loneliness on unemployment to persist throughout middle age, thus future investigation of causal mechanisms should not be restricted to younger populations. Given the detrimental effect of both loneliness and unemployment on health [26], and evidence of a relationship between loneliness and unemployment outside of their interaction with health, simultaneously addressing loneliness and unemployment has the potential to not only reduce healthcare burden and improve health outcomes, but improve economic prosperity and employability. This suggests benefit to integrated care not only within the healthcare sector (physical health, mental health, and social care), but also across sectors, including welfare and employment. Effort should be made to ensure individuals remain in employment, or where this is not possible provide support to prevent the transition to feeling lonely. Likewise, social security, social care and employers should be aware of the connection between loneliness and unemployment in order to prevent or manage any negative spiral that may arise from the onset of either experience.

Further causal analysis is required to consider the wider impact of loneliness on other economic, health and societal outcomes. Given this study’s novel identification of the direct impact of loneliness on unemployment, deeper insight into this pathway and potentially mediating factors is required. Now a relationship has been established between loneliness and unemployment, exploring potential mediation effects of additional health outcomes such as depression and anxiety would be of interest. Nevertheless, this study has identified the potential benefits of tackling loneliness and unemployment as precursors of both each other, and as previously suggested of health-related outcomes, thus preventing a potentially self-fulfilling negative cycle and limiting the burden to health, social care, employers and the welfare system. There is a need to improve understanding of the long-term vs short-term effects of loneliness and unemployment, and also to understand which subgroups of the population are more vulnerable to loneliness and subsequent unemployment. This, alongside the use of additional methods such as difference-in-difference, could be facilitated by continued collection of loneliness data to create longer data panels. Change in the definition of both loneliness (including ‘some of the time’) and unemployment (excluding ‘long-term sick and disabled’), while maintaining direction of change, reduced the impact of loneliness by around 50%. The sensitivity of our results suggest the need for further research into the mechanisms and risk factors surrounding this relationship. Further research should expand the horizon of the analysis to evaluate, for example, how the impact of changes in loneliness or unemployment evolves with time, or to explore the dynamic nature of unemployment and loneliness in a simultaneous longitudinal analysis of their apparent reinforcing and cumulative negative effects. This requires greater availability of data on loneliness, which will be increasingly prevalent as longitudinal studies, such as Understanding Society, include loneliness as core measures in data collection.

## Conclusion

Through application of causal methodology to both cross-sectional and longitudinal data, experience of loneliness is seen to have an even greater effect on unemployment than the more commonly studied reverse causal effect of unemployment on loneliness. Cross-sectional analysis reveals the impact of loneliness to be increasing over time rising from 16.0 percentage points in 2017–19 to 19.6 percentage points in 2018–20. Longitudinal analysis further reinforces findings revealing a 17.5 percentage point effect of loneliness on future unemployment, with particular impact from sustained experience of loneliness. The impact of loneliness on unemployment is exacerbated by interaction with work limiting physical health outcomes, and does not depend on physical health status. Overall, this study extends previous research and provides evidence of bidirectionality in the relationship between loneliness and unemployment across the UK working age population. In particular, loneliness is seen to impact both current and future unemployment suggesting efforts should be targeted at its prevention rather than treatment. Overall, improved loneliness could mitigate unemployment, and employment abate loneliness, which may in turn relate positively to other factors such as health and quality of life.

## Supplementary Information

**Additional file 1.**

## Data Availability

This paper uses data from Understanding Society, a UK Household Longitudinal Study. Understanding Society is an initiative funded by the Economic and Social Research Council and various Government Departments, with scientific leadership by the Institute for Social and Economic Research, University of Essex, and survey delivery by NatCen Social Research and Kantar Public. The research data are publicly available and distributed by the UK Data Service, http://doi.org/10.5255/UKDA-SN-6614-14.

## Abbreviations

PSM: Propensity score matching
ATE: Average treatment effect
