# Supplementary material for: Understanding the effect of loneliness on unemployment: propensity score matching
Source: BMC Public Health. 2022 Apr 28;22:740. doi: 10.1186/s12889-022-13107-x (PMC9045886; doi:10.1186/s12889-022-13107-x)
Supplement: Supplementary file 1 — Additional file 1. [file 12889_2022_13107_MOESM1_ESM.docx]

# **Appendix 1 – Supplementary Material**

***Table A1: Categories for unemployment status***

| **Employed** | **Unemployed** | **Exclude** |
| --- | --- | --- |
| Self-employed | Unemployed | Looking after family or home^Ϯ^ |
| In paid employment | Retired | Doing something else^Ϯ^ |
| Unpaid worker in family business | Long term sick or disabled | Full time studentᵟ |
| Working in apprenticeship |  | On government training scheme^Ϯ^ᵟ |
| On maternity leave |  |  |

^Ϯ^ unclear whether involves employment

ᵟ education

**Table A2: Binary coding for heterogeneity and subgroup probit regression**

| **Variable** | **Coded 0** | **Coded 1** |
| --- | --- | --- |
| Age | n/a included as a continuous variable in years | |
| Gender | Male | Female |
| Ethnicity | White | Non-white |
| Education | Higher degree | Other (lower) qualification |
| Marital status | Never married or not currently married | Married or in civil partnership |
| Household composition | One adult in the household | More than one adult in the household |
| Number of own children in household | No children | At least one child |
| Region | North, Midlands and devolved administrations (North East, North West, Yorkshire and Humber, East Midlands, West Midlands, Wales, Scotland, N. Ireland) | South (East of England, London, South East, South West) |
| How often feels lonely | Hardly ever or some of the time | Often |
| Current Economic activity | Employed | Unemployed |
| Health status (for reverse causal analysis) | Fair or poor | Excellent, very good or good |

**Table A3: Random effect probit regression models**

| **Regressors** | **Matched sample**  **Coefficient[95%CI]** | | **Propensity score weighted**  **Coefficient[95%CI]** | |
| --- | --- | --- | --- | --- |
|  | **Simple model** | **Full model** | **Simple model** | **Full model** |
| Constant | -7.812***  [-7.890,-7.735] | -6.948***  [-7.256,-6.640] | -6.378***  [-6.601,-6.156] | -7.435***  [-8.342,-6.528] |
| Loneliness | 0.837***  [0.616,1.058] | 1.158***  [0.997,1.319] | 0.907***  [0.355,1.458] | 1.070***  [0.557,1.583] |
| Gender |  | 0.199***  [0.081,0.317] |  | 0.039  [-0.367,0.444] |
| Age |  | 0.074***  [0.069,0.080] |  | 0.057***  [0.040,0.073] |
| Ethnicity |  | 0.921***  [0.766,1.075] |  | 1.113***  [0.615,1.611] |
| Education |  | 1.105***  [0.979,1.230] |  | 1.501***  [1.051,1.952] |
| Marital status |  | -0.771***  [-0.917,-0.624] |  | -0.693**  [-1.219,-0.167] |
| Household composition |  | -0.318***  [-0.483,-0.153] |  | -0.545*  [-0.994,-0.095] |
| Number own children in household |  | -1.309***  [-1.453,-1.165] |  | -1.027***  [-1.517,-0.536] |
| Region |  | -0.404***  [-0.526,-0.283] |  | -0.377  [-0.787,0.033] |

*p<0.05, **p<0.01, ***p<0.001. See TableA2 for variable definitions.

Models contain 38,400 observations in 22,725 groups.

***Figure A1: Heterogeneous Treatment Effect on Cross-Sectional Models***

Wave 9

Wave 10

*where lpoly smooth illustrates the pattern of treatment effect heterogeneity

# **Appendix 2 – Covariate balance**

**Table A4: Number of observations**

| **Model** | **Number of obs.** | | **Treated obs.** | | **Control obs.** | |
| --- | --- | --- | --- | --- | --- | --- |
|  | **Raw** | **Matched** | **Raw** | **Matched** | **Raw** | **Matched** |
| Model 1 | 19,566 | 39,132 | 1,626 | 19,566 | 17,940 | 19,566 |
| Model 2 | 18,833 | 37,666 | 1,526 | 18,833 | 17,307 | 18,833 |
| Model 3 | 15,675 | 31,350 | 1,854 | 15,675 | 13,821 | 15,675 |

**Table A5: Covariate balance before and after matching**

|  | **Model 1** | | | | **Model 2** | | | | **Model 3** | | | |
| --- | --- | --- | --- | --- | --- | --- | --- | --- | --- | --- | --- | --- |
| **Covariate** | **Standardised differences** | | **Variance ratio** | | **Standardised differences** | | **Variance ratio** | | **Standardised differences** | | **Variance ratio** | |
|  | **Raw** | **Matched** | **Raw** | **Matched** | **Raw** | **Matched** | **Raw** | **Matched** | **Raw** | **Matched** | **Raw** | **Matched** |
| Gender | 0.127 | -0.001 | 0.968 | 1.000 | 0.141 | 0.017 | 0.962 | 0.997 | 0.123 | -0.033 | 0.971 | 1.003 |
| Age | -0.245 | -0.026 | 1.133 | 0.993 | -0.289 | 0.014 | 1.110 | 1.017 | -0.283 | -0.028 | 1.146 | 1.006 |
| Ethnicity | 0.003 | 0.024 | 0.823 | 0.904 | 0.015 | -0.015 | 0.845 | 0.604 | 0.028 | 0.017 | 0.803 | 0.809 |
| Education | 0.096 | -0.008 | 1.076 | 0.894 | 0.065 | -0.000 | 1.054 | 0.950 | 0.088 | -0.011 | 1.089 | 0.909 |
| Marital status | 0.055 | 0.007 | 1.681 | 1.347 | 0.053 | 0.003 | 1.782 | 1.420 | 0.049 | -0.014 | 1.698 | 1.308 |
| Household composition | -0.020 | -0.025 | 1.295 | 1.170 | -0.059 | -0.023 | 1.307 | 1.223 | -0.007 | 0.020 | 1.306 | 1.206 |
| Number own children at home | -0.176 | 0.050 | 0.803 | 1.243 | -0.149 | 0.000 | 0.905 | 1.179 | -0.125 | 0.025 | 0.919 | 1.218 |
| Region | -0.065 | 0.000 | 0.956 | 0.933 | -0.024 | -0.031 | 0.989 | 0.973 | -0.074 | -0.024 | 0.976 | 1.000 |

***Figure A2: Balance in Raw and Matched Samples***

Model 1

Model 2

Model 3
